# Supplementary material for: Autophagy Controls Sulphur Metabolism in the Rosette Leaves of Arabidopsis and Facilitates S Remobilization to the Seeds
Source: Cells. 2020 Jan 31;9(2):332. doi: 10.3390/cells9020332 (PMC7073174; doi:10.3390/cells9020332)
Supplement: Supplementary file 1 [file cells-09-00332-s001.pdf]

Supplemental Fig. S1

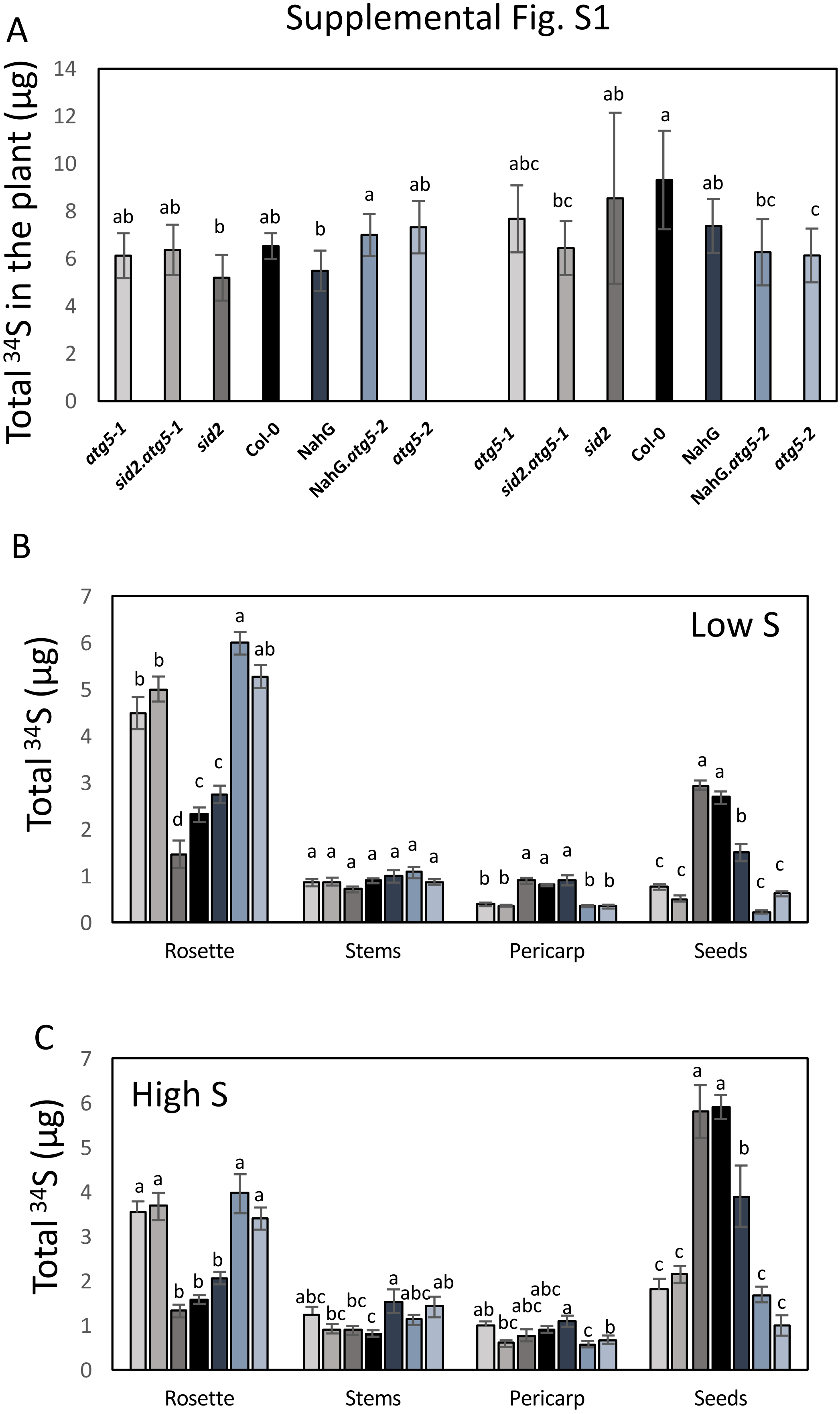

Figure S1: Total ^{34}S content in *atg5* autophagy mutants and control lines under both Low S and High S. The ^{34}S content (µg) in the whole plant (A) and in individual organs (rosette, stem, pericarp and seeds) of *atg5* mutants and control lines were measured (B, Low S; C, high S). The different letters indicate values significantly different at $P<0.05$ (n=12) as determined using ANOVA Newman-Keuls (SNK) comparison.
